# Self-Healing Hydrogel Scaffolds through PET-RAFT Polymerization in Cellular Environment

**DOI:** 10.1021/acs.biomac.3c00431

**Published:** 2023-06-29

**Authors:** Alasdair D. M. Rigby, Amaziah R. Alipio, Viviane Chiaradia, Maria C. Arno

**Affiliations:** † School of Chemistry, University of Birmingham, Edgbaston, Birmingham B15 2TT, U.K.; ‡ Institute of Cancer and Genomic Sciences, University of Birmingham, Edgbaston, Birmingham B15 2TT, U.K.

## Abstract

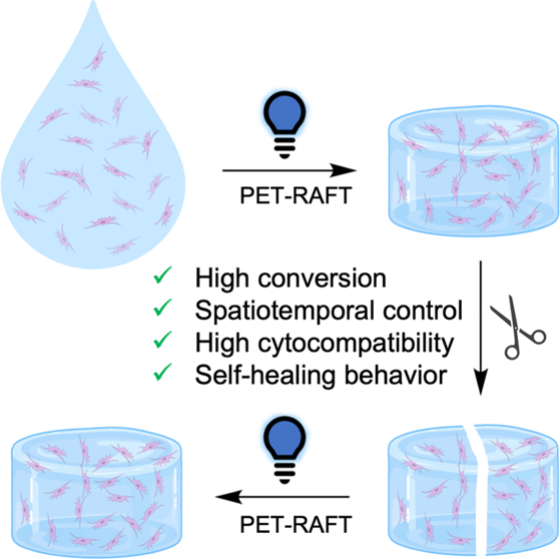

Photo electron/energy transfer-reversible addition–fragmentation chain transfer (PET-RAFT) has emerged as a powerful reversible-deactivation radical polymerization technique, enabling oxygen-tolerant polymerizations with exquisite spatiotemporal control through irradiation with visible light. In contrast to traditional free radical photo-polymerization, which often requires the use of DNA-damaging UV irradiation, PET-RAFT offers a more cytocompatible alternative for the preparation of polymeric materials in cell culture environments. Herein, we report the use of PET-RAFT for the fabrication of self-healing hydrogels using commercially available monomers, reaching high monomer conversions and cell encapsulation efficiencies. Our hydrogels showed the expected rheological and mechanical properties for the systems considered, together with excellent cytocompatibility and spatiotemporal control over the polymerization process. Moreover, hydrogels prepared through this method could be cut and healed again by simply adding further monomer and irradiating the system with visible light, even in the presence of mammalian cells. This study demonstrates for the first time the potential of PET-RAFT polymerization as a viable methodology for the synthesis of self-healing hydrogel scaffolds for cell encapsulation.

## Introduction

Since its first report in 2014, photo electron/energy transfer-reversible addition–fragmentation chain transfer (PET-RAFT) polymerization has significantly advanced the field of reversible-deactivation radical polymerization (RDRP), allowing access to a diverse range of polymer architectures in the presence of oxygen and in cytocompatible media, while achieving excellent spatiotemporal control over the polymerization process.^1−6^ Although transition metal catalysts, such as *fac*-[Ir(ppy)_3_], Ru(bpy)_3_Cl_2_, and ZnTPP, were initially used to obtain polymers with high monomer conversion and low dispersity,^1,7,8^ organic photo-catalysts represent a more attractive option owing to their higher cytocompatibility and commercial availability.^9−11^ Among these, eosin Y (EY) has received wide attention, allowing for well-defined polymeric structures to be synthesized within both organic and aqueous environments at low catalyst loading (10 ppm).^9,12−15^ Moreover, EY has demonstrated high cytocompatibility, having been used as a photo-catalyst for polymerizing water-soluble monomers from the surface of eukaryotic cells.^16,17^

More recently, PET-RAFT polymerization has been used for the preparation of crosslinked materials.^18−20^ In comparison to other photo-polymerization strategies, the PET-RAFT approach has been reported to enable superior control over polymer growth and excellent uniformity of the resultant polymer networks, by providing an additional pathway for radical deactivation.^21^ As a result, not only was the overall dispersity of the crosslinked systems lowered but the materials also showed improved swelling properties. Further advances in this field have also seen the development of 3D printing PET-RAFT techniques, enabling spatiotemporal control over the 3D printing process and achieving materials that can be further functionalized post-printing.^20,22^

Among 3D crosslinked materials, hydrogels have gained significant interest over the last decade as soft tissue scaffolds, as a consequence of their high water content, cytocompatibility, and tunable mechanical properties.^23−26^ These materials have demonstrated great promise for cell encapsulation as they can mimic the structural composition and mechanical properties of native extra-cellular matrix,^27,28^ enabling increased cell viability over prolonged time and promoting stem cell differentiation.^24^ The fabrication of hydrogel networks directly from monomers in the presence of cells has been exploited using free radical photo-polymerization (FRP) processes that require the use of UV irradiation^24,29−34^ or visible light.^35^ However, UV irradiation is well known for its poor cytocompatibility, long-term DNA damage, and limited penetration to biological tissues, making it a less-than-ideal choice for the fabrication of biomaterials.^10,36−39^ Typical photo-initiators excited by exposure to UV light are cytotoxic and not soluble in water, further limiting the use of FRP in biologically relevant applications.^40^ Furthermore, free radical processes have been shown to afford less uniform networks, as a consequence of the increased termination reactions, reducing the properties of the resultant materials such as swelling ratio and mechanical performance.^41,42^ RDRP techniques, such as atom transfer radical and RAFT polymerizations have been used to improve material properties by accessing precise polymer architectures and low dispersities, which, in turn, result in more uniform polymer networks.^41,43^

Herein, we hypothesized that PET-RAFT polymerization could be used to generate hydrogel scaffolds directly in phosphate buffer saline (PBS) and cell culture media, using a cytocompatible photocatalyst (EY) through exposure to visible light (450 nm). Exploiting a series of commercially available monomers, high monomer conversions (>90%) could be reached with the formation of soft cellular scaffolds, while retaining the spatiotemporal control over the polymerization characteristic of PET-RAFT. Hydrogels prepared through this method demonstrated excellent cytocompatibility, with the polymerization process and exposure to visible light being well tolerated by murine progenitor liver cells. Finally, owing to the controlled nature of the PET-RAFT technique and the presence of chain transfer agents at the end of the polymerized chains, hydrogels could be cut and, subsequently, healed in the presence of encapsulated cells.

## Experimental Section

### Materials

4-cyano-4-{[(ethylthio)carbonothioyl]thio}pentanoic acid (CEPA) was synthesized following a previously reported method.^44^ Poly(ethylene glycol) methacrylate (average *M*_n_ 360 Da), poly(ethylene glycol) diacrylate (average *M*_n_ 575 Da), *N*,*N*-dimethylacrylamide, 2-(dimethylamino)ethyl methacrylate, *N*,*N*-methylene-bis-acrylamide, deuterium oxide, potassium phthalate monobasic, and eosin Y were purchased from Sigma-Aldrich. Liquid monomers were passed through aluminum oxide before use to remove the inhibitor. Aluminum oxide was purchased from Acros Organics. Phosphate buffered saline tables were purchased from Thermo Scientific and the PBS solution was prepared fresh in deionized or deuterium oxide water upon use. Murine hepatic progenitor cells (HPCs) were kindly donated by Ms Melissa Vieira (University of Birmingham). Dulbecco’s modified eagle medium (DMEM), penicillin–streptomycin, and l-glutamine were purchased from Gibco. Fetal bovine serum (FBS) and sterile PBS were purchased from Sigma-Aldrich. Live/Dead viability/cytotoxicity kit was purchased from Invitrogen.

### Instrumental and Analytical Methods

#### NMR Spectroscopy

^1^H NMR spectra were recorded on a Bruker 400 MHz spectrometer at 298 K. Spectra were analyzed using the MestReNova software.

#### Photo-Rheology

Rheological analysis was carried out on an Anton Parr MCR 302 rheometer for real-time photocuring. The setup comprised a detachable photoillumination system [OmniCure S1500 curing system with a 400–500 nm filter (14.5 W cm^–2^), broadband Hg-lamp and a glass plate]. Time sweep tests were performed to investigate storage and loss modulus changes over time. Measurements were taken using a 30 mm parallel plate at 25 °C, with frequency and strain of 0.5 Hz and 1%, respectively. Data were processed through the RheoCompass software. Mechanical testing and compression mechanical analysis was carried out on a Testometric M350-5CT with a 5 kgf load cell. Analysis was carried out using the WinTest Analysis software.

### Hydrogel Synthesis

Stock solutions of CEPA and EY (100 and 1 mg mL^–1^, respectively) were prepared in acetone. Specific amounts (depending on hydrogel formulation, see Table S1) were aliquoted from the stock solutions and dried under a flow of nitrogen. Liquid monomers were passed through a column of basic alumina before the subsequent addition of both the CEPA and EY into the mixture diluted with PBS (500 μL). *N*,*N*-Methylene-bis-acrylamide (NMBA) was added without further purification. Exact quantities of each reagent used for each hydrogel formulation can be found in the Supporting Information (Table S1). The resulting solution was taken up into a 2 mL syringe and lowered into a lightbox for irradiation (Figure S1). The solution was irradiated with blue light (450 nm, 11.5 W) for 1 h, during which the temperature inside the photoreactor reaches a maximum of 35 °C. The formed hydrogel was then removed from the syringe, washed with D_2_O (500 μL) to remove any unreacted monomer (this solution was further used to measure monomer conversion), and stored in the fridge for further analysis.

### Hydrogel Self-Healing

To conduct self-healing experiments, the prepared hydrogels were cut in half using a scalpel. The two-halves of hydrogel were then placed in proximity of each other, though avoiding contact, and additional monomer (approximately 100 μL, enough to fill the gap) was added. The hydrogel was then irradiated with blue light (450 nm, 11.5 W) for 1 h before washing with PBS to remove any unreacted monomer, leaving the healed structure for further characterization.

### Hydrogel Characterization

#### Monomer Conversion (*c*)

The D_2_O solution used for hydrogel washing, containing unreacted monomer, was mixed with 200 μL of a potassium phthalate monobasic (PHP) stock solution (50 mg mL^–1^ in D_2_O). The resulting solution containing the standard was then analyzed *via* quantitative ^1^H NMR spectroscopy (qNMR) to determine the quantity of unreacted monomer by comparing the integrals of the peaks corresponding to the monomer (*I*_x_) to those of the internal standard (*I*_cal_). By comparing the number of protons associated with the monomer (*N*_x_), internal calibrant (*N*_cal_), and the concentration of internal calibrant present (*C*_cal_), the concentration of monomer (*C*_x_) could be calculated for each hydrogel using eq 1. Analysis was carried out in triplicate (*N* = 3). In the spatiotemporal control experiments, PHP was found to acidify the solution and lower the activity of EY,^45^ as such a correction factor was applied to calculate monomer conversion (5.13× for PEGDA, 2.67× for PDMA-NMBA).
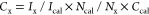
1

#### Equilibrium Water Content

The hydrogels’ swelling properties were characterized by measuring their equilibrium water content (EWC), which is a measure of the quantity of water able to be retained in the hydrogel network. Hydrogels were immersed in PBS (pH 7.4) for 24 h at 37 °C, dried gently with a paper towel, and their weight recorded (*W*_s_). They were then lyophilized, and their weight recorded again (*W*_d_). The EWC of each hydrogel was calculated using eq 2. Analysis was carried out in triplicate (*N* = 3).
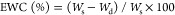
2

#### Swelling Factor

Hydrogels were fabricated as described previously and left to cure for 2 h at room temperature. The prepared hydrogels were then placed in PBS solution pH 7.4 and incubated at 37 °C. The PBS solution was replaced regularly to remove unreacted monomer precursors. At set time intervals, the hydrogels were removed, gently dried, and their weight recorded. The swelling factor (SF) was calculated using eq 3, where *W*_t_ is the weight of the hydrogel at each time point, and *W*_0_ is the initial wet hydrogel weight (before swelling). Analysis was carried out in triplicate (*N* = 3).
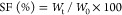
3

### Photo-Rheology

For rheological characterization, a solution containing monomer, CEPA, and EY (0.1 mL) was placed between two parallel plates. A small amount of PBS was added around the lower plate to prevent drying and a temperature chamber was used to keep the temperature constant at 25 °C. The mixture was sheared at 0.5 Hz and 1% of amplitude for 1 min without light irradiation. After this, the light source was switched on and the changes on storage and loss modulus were investigated.

### Mechanical Testing

For mechanical testing, freshly made hydrogels were washed with PBS (2 mL) and stored for 2 h. Hydrogels were then dried gently with a paper towel, and their height and diameter recorded. Hydrogels were placed between the tensiometer heads and a preload force of 0.1 N was set. Tests were then carried out at a compression velocity of 3 mm min^–1^. Analysis was carried out with Young’s modulus and strain at break and stress at break calculated from the average of minimum 6 repeats. The first 1% of strain was used to calculate the Young’s modulus values.

### Cell Encapsulation and Viability Measurements

HPCs were grown in 75 cm^2^ cell culture flasks that were pre-coated overnight with 200 μg mL^–1^ type-1 rat tail collagen. Cells were maintained by renewing culture medium every 3–4 days and passaging every 7 days or upon reaching 90% confluency. DMEM cell culture media was supplemented with 10% fetal bovine serum, 100 units mL^–1^ penicillin, 100 μg mL^-1^streptomycin, and 2 mM l-glutamine. Cells were detached from cell culture flasks using 0.25% trypsin to obtain a concentrated cell suspension that was re-suspended in DMEM. For each gel, cells were gently mixed with defined amounts of CTA, EY, and monomers (Table S2) to reach a total volume of 500 μL and a final cell density of 3 × 10^6^ cells mL^–1^. From this mixture, 200 μL was taken up into a 2 mL syringe and polymerized by irradiation with blue light (450 nm, 11.5 W) for 20 min. The resulting cell-laden hydrogels were washed with DMEM and left to recover in humidified atmosphere overnight (37 °C, 5% CO_2_). Cell viability was determined using a Live/Dead viability/cytotoxicity kit purchased from Invitrogen. The cell-laden hydrogels were placed in PBS containing calcein and ethidium homodimer as live/dead strains, following the manufacturer’s protocol, for at least 1 h. Hydrogels were then washed with fresh PBS and imaged using an Olympus Fluoview FV3000 microscope. Live/dead cells were counted using the Image J Cell Counter plugin and viability was determined as a percentage of live cells over total cell number. At least 3 representative Z-stack images were obtained (10× magnification) with a capture depth >500 μm. Cell-laden hydrogels were left to incubate in fresh DMEM for a further 7 days, at which the viability assay was repeated to obtain day 7 images.

### Self-Healing of Cell-Laden Hydrogels

Self-healing was performed as previously described. Briefly, the hydrogel was cut in half such that a gap was present. A mixture consisting of 3:10 cell suspension to monomer was added to the gap and the gel was irradiated with blue light (450 nm, 11.5 W) for 20 min. The healed gels were washed with DMEM to remove unreacted monomer. The healed, cell-laden hydrogel was incubated overnight (37 °C, 5% CO_2_), and cell viability was assessed as described earlier.

## Results and Discussion

### Hydrogel Synthesis

**Figure 1:**
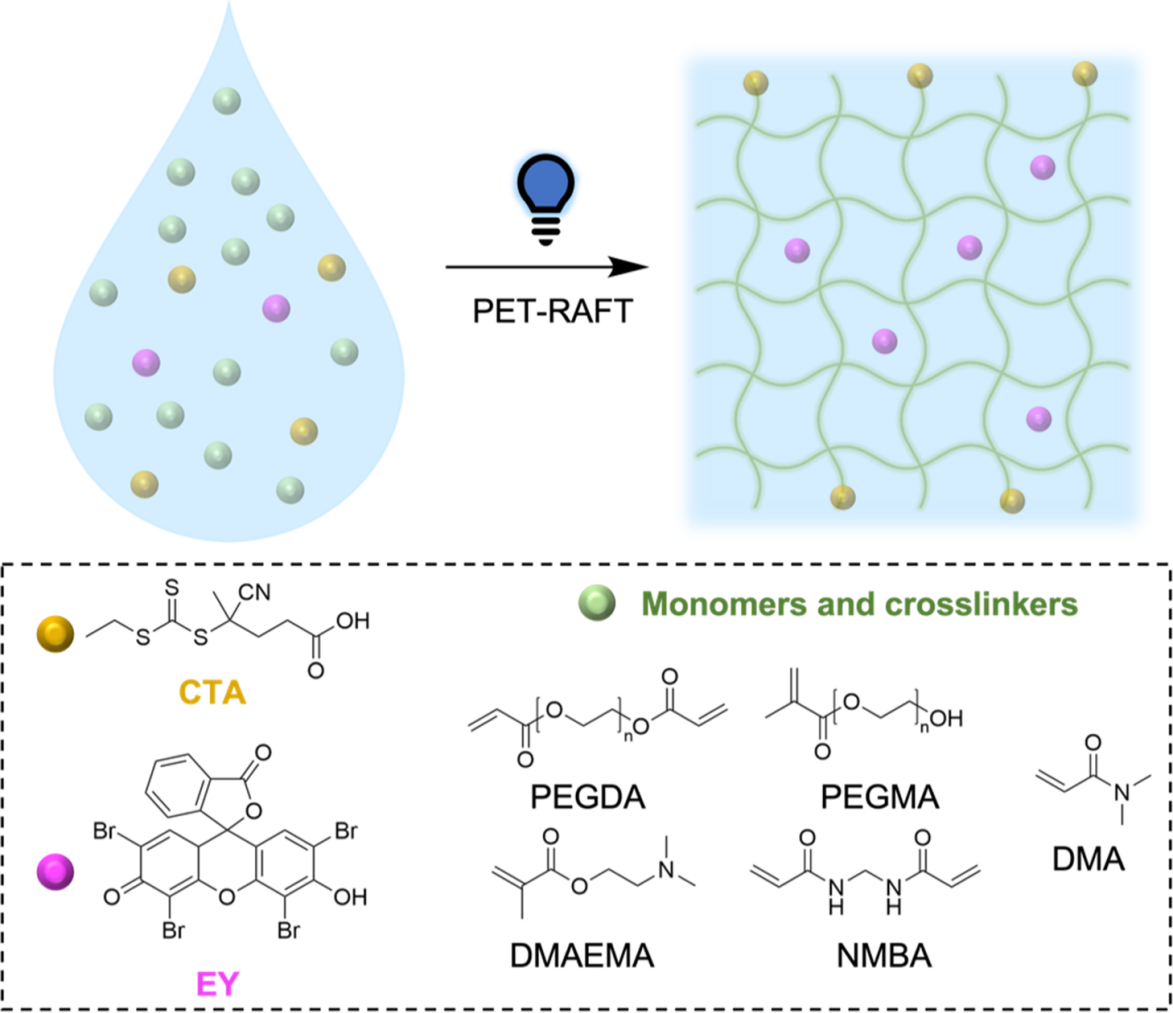
PET-RAFT hydrogel systems used in this work. Monomers and crosslinkers are combined with the CTA and EY in PBS and irradiated with 450 nm blue light to produce 3D hydrogel networks.

In order to obtain hydrogel scaffolds relevant for cell encapsulation, all monomers selected for this project were polymerized directly in PBS, using the cytocompatible EY as a catalyst and 4-cyano-4-{[(ethylthio)carbonothioyl]thio}pentanoic acid (CEPA) as the chain transfer agent (CTA) (Figure 1). A range of commercially available (meth)acrylate and acrylamide monomers, i.e., poly(ethylene glycol) diacrylate (PEGDA), poly(ethylene glycol) methacrylate (PEGMA), *N*,*N*-dimethylacrylamide (DMA), 2-(dimethylamino)ethyl methacrylate (DMAEMA), and *N*,*N*-methylene-bis-acrylamide (NMBA) were selected for investigation for their water solubility and their reported ability to form crosslinked networks.^46−49^ Polymerizations were carried out inside a plastic syringe, using a typical photoreactor setup for PET-RAFT (Figure S1). All polymerizations were carried on for 60 min to ensure full gelation was achieved.

The obtained hydrogels were washed with D_2_O to recover unreacted monomer, and the solution was mixed with a known amount of potassium phthalate monobasic (PHP) as an internal standard. Monomer conversions were determined using ^1^H qNMR spectroscopy by comparing the integrals of the peaks corresponding to the residual monomer to those of the internal standard (Table 1 and Figures S2–S5). Although monomer conversions reached above 90% for all systems considered, it is worth noting that this only reflects the amount of monomer consumed, without being a direct measure of reacted or unreacted double bonds. The hydrogels’ swelling properties were then characterized by measuring their EWC after immersion in PBS at 37 °C for 24 h (Table 1).

**Table 1:** Hydrogel Formulations Investigated in This Work along with Monomer Conversions (C) Measured for Each System and EWC in PBS

hydrogel formulation^a^	targeted DP	CTA/monomer/crosslinker/EY ratio	C^b^ (%)^c^	EWC (%)^c^
PEGDA	50	1/50/0/0.00565	91 ± 1.9	85 ± 0.2
PEGDA	100	1/100/0/0.00565	95 ± 0.4	82 ± 0.2
PEGMA-PEGDA (5 wt %)	100	1/100/3.1/0.00565	100 ± 0.1	89 ± 1.1
PEGMA-PEGDA (10 wt %)	100	1/100/6.2/0.00565	100 ± 0.1	87 ± 0.5
PDMAEMA-PEGDA (50 wt %)	100	1/100/13.7/0.00565	100 ± 0.1	82 ± 0.1
PDMA-NMBA (30 wt %)	100	1/100/19.3/0.00565	93 ± 0.6	87 ± 1.2
PDMA-NMBA (50 wt %)	100	1/100/32.1/0.00565	95 ± 0.8	82 ± 0.3

a The percentage in brackets refers to the amount of crosslinker used in relation to monomer. 0.5 mL of PBS were added to each formulation.

b Monomer conversion was calculated *via* qNMR spectroscopy against potassium phthalate monobasic (PHP) as an internal standard.

c Values arereported as average ± standard deviation (*N* = 3).

First, PEGDA hydrogels were prepared with a degree of polymerization (DP) of 50 and 100. In both cases, PEGDA was polymerized with high conversions (91 ± 1.9% and 95 ± 0.4% for DP 50 and 100, respectively) (Table 1, Figure S2) with the polymer targeting a higher DP reaching higher conversions as a consequence of less deactivation of the CTA and increased propagation.^50^ For the PEGDA DP 50 system, conversion could be further improved (up to 96%) by increasing EY concentration from 30 to 51 ppm (Table S3), demonstrating the ability to tune molecular weight and, hence, degree of crosslinking. Nevertheless, the amount of EY required for the polymerizations is within the cytocompatible range and far below the typical photo-initiator concentrations (1000–5000 ppm) used for the synthesis of PEGDA hydrogels under UV light.^51,52^ Independently from the concentration of EY used, PEGDA hydrogels were confirmed to have good swelling properties, as evinced by their capability to retain water within the hydrogel network (EWC > 80%), which is expected for hydrogels prepared from this monomer (Table 1).^29^

In order to expand the synthesis of hydrogel scaffolds to mono-substituted monomers, PEGMA was co-polymerized with the bi-functional crosslinker PEGDA. Two hydrogel sets were prepared, both with PEGMA (DP 100) and different amounts of PEGDA crosslinker, 5 and 10 wt %, respectively. Both hydrogels reached 100% conversion after 60 min (100 ± 0.1% and 100 ± 0.1%, respectively) (Table 1, Figure S3), confirming the great potential of PET-RAFT polymerization for the synthesis of hydrogel scaffolds. Both hydrogels also have similar swelling properties (89 ± 1.1 and 87 ± 0.5% for samples containing 5 and 10 wt % of crosslinker, respectively) (Table 1), suggesting that the higher amount of crosslinker has little impact on swelling properties at high monomer conversions.

To expand the scope of our work beyond PEG-based monomers, the ability of other water-soluble monomers, such as DMAEMA and DMA, to form hydrogel networks in the presence of a bi-functional crosslinker was also investigated. PDMAEMA (DP 100) was crosslinked with PEGDA, with hydrogels obtained only when 50 wt % of crosslinker was added, likely as a consequence of the high solubility in water of the cationic polymer. PDMAEMA-PEGDA hydrogels reached 100% conversion (Table 1, Figure S4) and exhibited swelling properties comparable to the other systems. In contrast to all other hydrogels, which retained a pink color owing to the presence of EY trapped within the 3D network, PDMAEMA-PEGDA hydrogels appeared yellow in color (Figure S6d), which is typically observed for PDMAEMA polymers.^53−55^

Finally, to explore a different system, PDMA hydrogels crosslinked with NMBA were investigated. Initial screenings showed that a minimum of 30 wt % NMBA was necessary for hydrogel formation within the 60 min of irradiation considered in this study. As such, PDMA-NMBA hydrogels with 30 and 50 wt % NMBA crosslinker were prepared. For both systems, similar conversions were achieved (93 ± 0.6 and 95 ± 0.8% for 30 and 50 wt % NMBA, respectively) (Table 1, Figure S5). As observed for the previous hydrogels, the increase in crosslinker concentration did not significantly affect swelling properties, with the hydrogel at 50 wt % NMBA displaying an EWC of 82 ± 0.3% and the hydrogel at 30 wt % exhibiting an EWC of 87 ± 1.2%, within their expected ranges (Table 1).^56^

To assess the swelling ability of our hydrogels prepared through the PET-RAFT approach, the swelling factor (SF) was measured as a function of time (Figure S7). The PDMAEMA-PEGDA (50 wt %) hydrogel was the only system that swelled considerably, reaching a SF of 194 ± 0.8% after 72 h. A slight swelling could also be observed for both PEGMA-PEGDA systems (5 and 10 wt %), although less significant than the swelling observed for PDMAEMA-PEGDA crosslinked hydrogels. Interestingly, both PEGDA hydrogels (DP 50 and DP 100), as well as both PDMA-NMBA systems (30 and 50 wt %), slightly shrunk within 24 h of being immersed in PBS solution at 37 °C. We hypothesize that this behavior may be a consequence of the more hydrophobic nature of the hydrogel networks and lower conversions of the PEGDA and PDMA-NMBA systems, which translate in these hydrogels repelling water out, therefore decreasing their weight.

### Photo-Rheology

**Figure 2:**
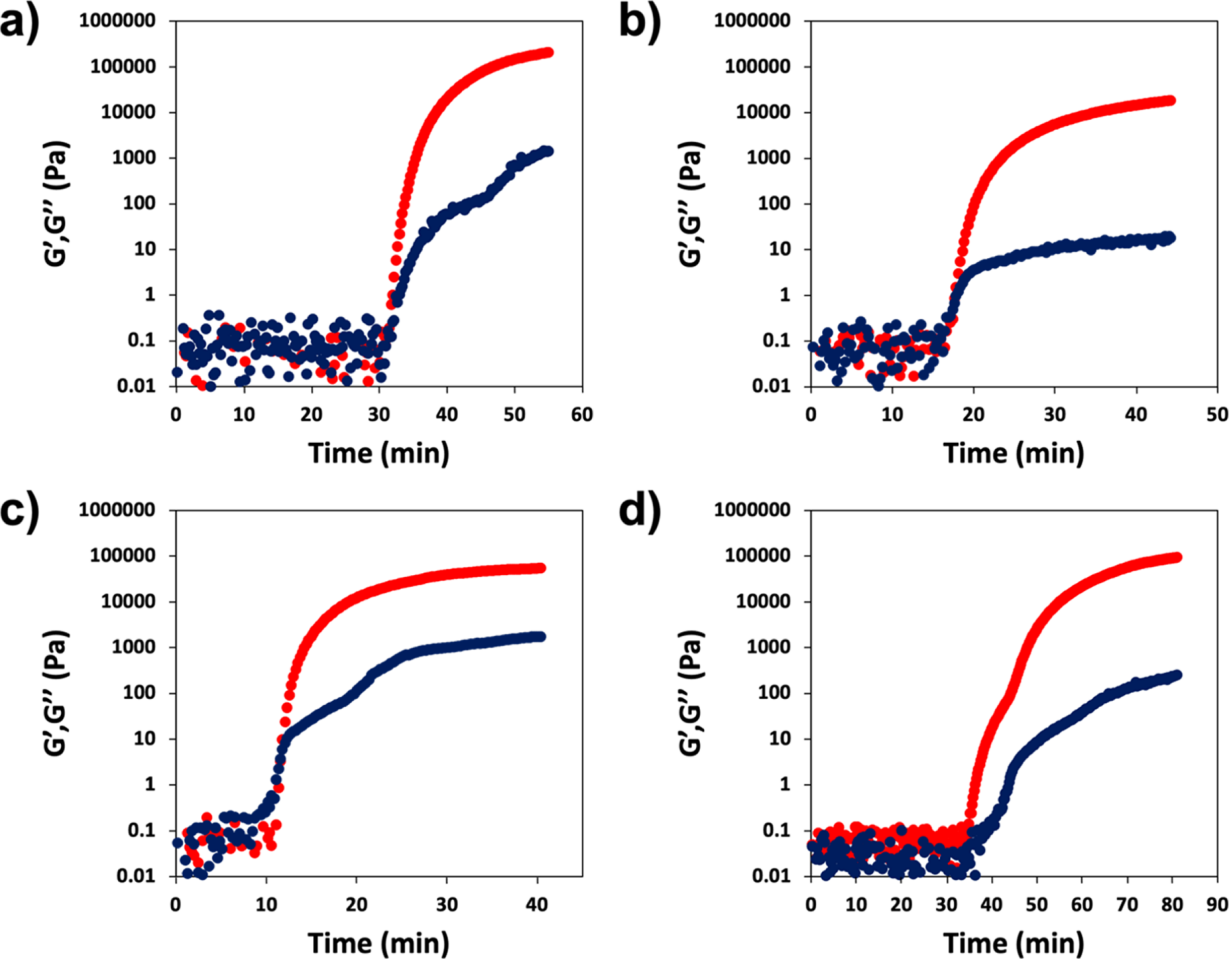
Photo-rheology of (a) PEGDA (DP 50), (b) PEGMA-PEGDA (5 wt %), (c) PDMAEMA-PEGDA (50 wt %), and (d) PDMA-NMBA (30 wt %) hydrogels showing the storage modulus (*G*′, red line) and loss modulus (*G*″, blue line) vs irradiation time.

Real-time rheology was carried out for each hydrogel system to determine their gelation kinetics, by monitoring the intersection between the storage modulus (*G*′) and the loss modulus (*G*″) after irradiation with visible light (400–500 nm) (Figures 2 and S8, Table 2). To carry out photo-rheological experiments, solutions of monomer, crosslinker, CTA, and EY were placed in the rheology plates while being irradiated with blue light (400–500 nm) to measure changes in storage and loss moduli over time. PEGDA hydrogels were investigated first, with the hydrogel with a targeted DP of 100 reaching gelation over 20 min faster when compared to the PEGDA hydrogel with a targeted DP of 50, with measured gelation times of 8 and 31 min, respectively (Figure 2a and S8). Moreover, the PEGDA hydrogel at DP 100 displayed superior strength in comparison to PEGDA DP 50, exhibiting a *G*′ of 4.63 × 10^5^ Pa in comparison to 2.09 × 10^5^ Pa.

**Table 2:** Gelation Times and *G*′ Maximum Values Obtained from Photo-Rheology Experiments and Mechanical Testing Analysis for Hydrogels Prepared in This Work, Showing Average Compressive Young’s Modulus, Strain at Break, and Stress at Break

hydrogel formulation^a^	gelation time (min)	G′ max (Pa)	Young’s modulus, *E* (kPa)	strain at break (%)	stress at break (kPa)
PEGDA (DP 50)	31	2.09 × 10^5^	183.4 ± 6.3	11.2 ± 1.7	72.7 ± 30.9
PEGDA (DP 100)	8	4.63 × 10^5^	406.5 ± 10.5	7.9 ± 1.1	53.3 ± 29.3
PEGMA-PEGDA (5 wt %)	17	1.08 × 10^4^	145.2 ± 9.1	13.6 ± 1.2	48.0 ± 11.0
PEGMA-PEGDA (10 wt %)	21	1.16 × 10^4^	69.9 ± 3.1	14.2 ± 2.2	28.0 ± 12.7
PDMAEMA-PEGDA (50 wt %)	11	5.46 × 10^4^	155.4 ± 19.1	15.2 ± 1.4	117.0 ± 25.2
PDMA-NMBA (30 wt %)	35	9.40 × 10^4^	177.3 ± 17.9	9.1 ± 1.6	31.3 ± 14.2
PDMA-NMBA (50 wt %)	41	5.73 × 10^4^	557.4 ± 45.0	5.2 ± 1.9	54.0 ± 27.9

a The percentage in brackets refers to the amount of crosslinker used in relation to monomer. Values are reported as average ± standard deviation, where *N* = minimum 6.

PEGMA-PEGDA hydrogels reported gelation points within a similar time range to PEGDA DP 100 hydrogels, with gelation times of 17 and 21 min for hydrogels with 5 and 10 wt % of bi-functional crosslinker, respectively (Figures 2b and S8). As expected, both hydrogels exhibited similar values of *G*′ (1.01 × 10^4^ and 1.16 × 10^4^ Pa at 5 and 10 wt % PEGDA, respectively), demonstrating that a small increase in the amount of crosslinker has little effect on the resulting hydrogel properties.

The hydrogel obtained from PDMAEMA-PEGDA gelled in only 11 min (Figure 2c), demonstrating that gelation is achievable in under 60 min, though longer irradiation time is likely required to achieve the storage modulus plateau. A *G*′ of 5.46 × 10^4^ Pa was obtained for this system, slightly higher than that found for PEGMA-PEGDA hydrogels.

Finally, PDMA-NMBA systems displayed the longest gelation times (35 and 41 min for 30 and 50 wt % of NMBA, respectively) (Figure 2d and S8). An increase in crosslinker concentration did not significantly affect hydrogel strength, with the hydrogel containing 30 wt % NMBA showing a *G*′ of 9.40 × 10^4^ Pa and the one containing 50 wt % NMBA exhibiting a *G*′ of 5.73 × 10^4^ Pa.

To further evaluate the mechanical strength of our hydrogels prepared through the PET-RAFT approach, uniaxial compressive tests were undertaken to determine the ultimate compressive stress and Young’s modulus for each system. The hydrogels were synthesized in syringes and then left to cure for 2 h before compression tests were carried out and the resultant stress and strain were measured (Table 2 and Figure S9). All strain at break values were found similar among the hydrogel systems observed, with the PDMA-NMBA (50 wt %) hydrogels rupturing at the lowest strain (5.2%), and the PDMAEMA-PEGDA (50 wt %) hydrogels displaying the highest strain (15.2%). Similarly, no significant difference was observed among the stress at break values, ranging from 28 to 117 kPa for all hydrogels. Interestingly though, the PEGDA DP 50 system presents a slightly higher stress at break compared to the PEGDA DP 100 (72.7 *vs* 53.3 kPa), although the latter displays a higher Young’s modulus (183.4 kPa for PEGDA DP 50 *vs* 406.5 kPa for PEGDA DP 100). Although the PEGDA DP 50 hydrogels could not spring back to their original shape once the force was released, they did not break under the applied force. On the contrary, PEGDA DP 100 hydrogels showed a more brittle structure, likely as a consequence of the higher degree of crosslinking, which, in turn, results in a higher Young’s modulus.^57^ PEGDA (DP 50) hydrogels prepared with a higher EY concentration displayed an increased Young’s modulus and stress at break (360.9 and 138.2 kPa, Table S4), likely as a consequence of the higher conversion of this system and higher initiation rate. Nevertheless, rheological and mechanical properties of hydrogels prepared through the PET-RAFT approach are similar to those of hydrogels prepared through free radical polymerization for the monomers and crosslinkers considered.^58−61^

### Spatial and Temporal Control

**Figure 3:**
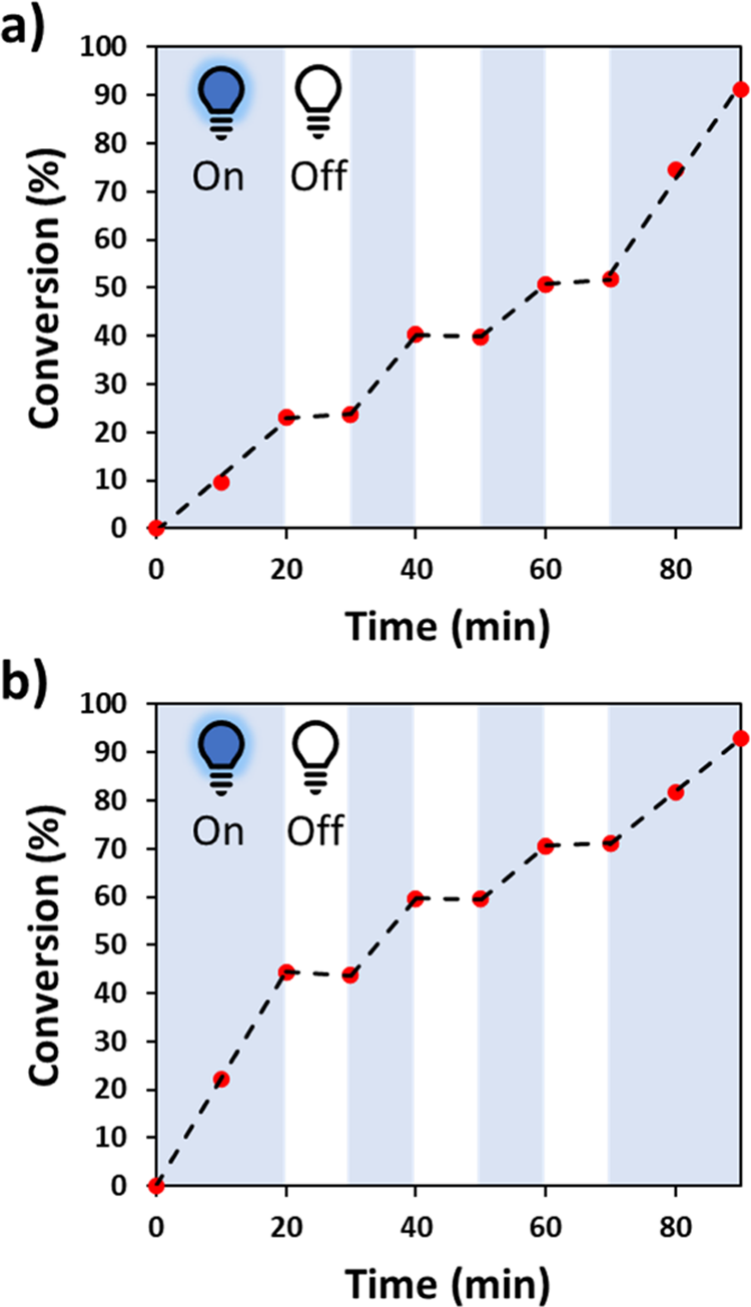
Spatiotemporal control observed during PET-RAFT polymerizations. Graphs show the monomer conversion of (a) PEGDA (DP 50) and (b) PDMA-NBMA (30 wt %) when irradiation is present (on) and absent (off).

PET-RAFT has previously been shown to exhibit excellent spatiotemporal control over the polymerization process, owing to the deactivation of the photo-catalyst when irradiation is stopped and subsequent reactivation of the polymerization when the light is switched back on.^1,7,62,63^ To demonstrate this effect toward the synthesis of our hydrogel materials, two of our hydrogel formulations, PDMA-NMBA (30 wt %) and PEGDA (DP 50), were selected as representative systems for this study. PDMA-NMBA (30 wt %) and PEGDA (DP 50) solutions were prepared with the addition of EY and CTA. PHP was added directly as internal calibrant to each. The solutions were then irradiated for periods of 10 min, followed by 10 min in the dark, with monomer conversion determined *via*^1^H qNMR spectroscopy. NMR spectroscopy analysis revealed that monomer conversion was achieved with irradiation, while no significant monomer conversion was achieved when the sample was kept in the dark, demonstrating the spatiotemporal control of this process in the formation of 3D hydrogel materials (Figure 3).

### Cell Encapsulation and Cytocompatibility

**Figure 4:**
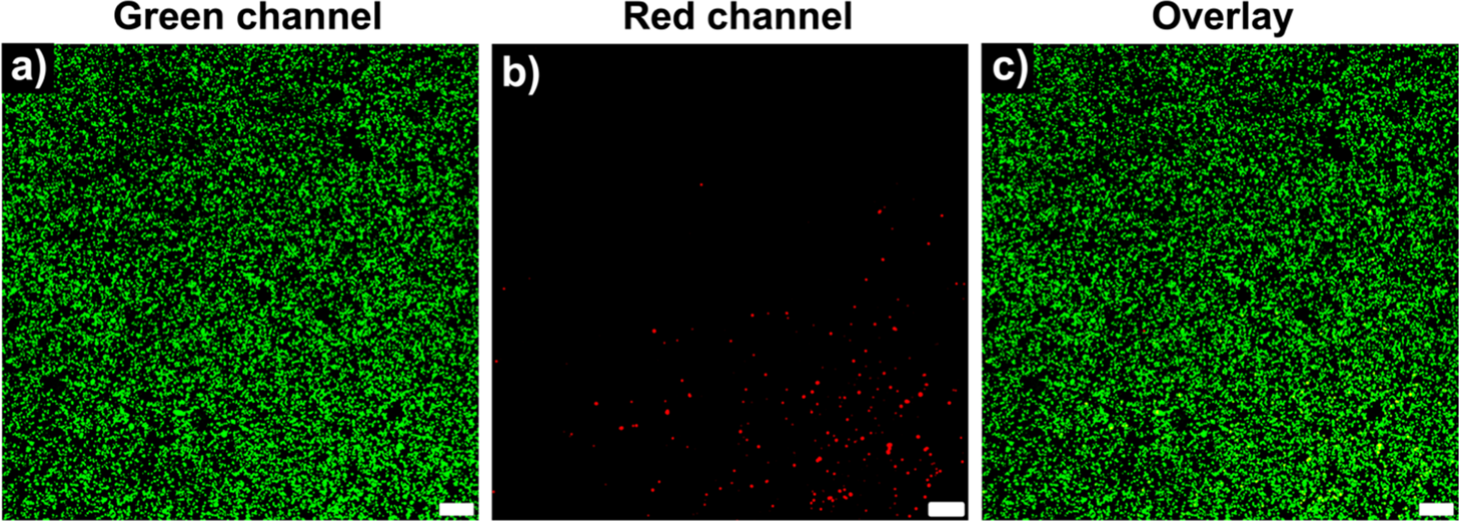
Representative confocal z-stacks (680 μm depth) of HPCs 24 h after encapsulation in PEGDA (DP 50) hydrogels. Cell-laden hydrogels were maintained in DMEM supplemented with 10% FBS and was stained with a Live/Dead viability staining kit to indicate (a) live cells (green channel), (b) dead cells (red channel), and (c) overlay of green and red channels. Scale bar = 200 μm.

In order to encapsulate living mammalian cells, hydrogels were prepared directly in cell culture media. Hepatic progenitor cells (HPCs) were selected as an ideal cell line for encapsulation, as they are unable to adhere to tissue culture plates and require to be encapsulated in 3D materials or seeded on a collagen layer to survive and proliferate.^64^ PEGDA (DP 50), PEGMA-PEGDA (5 wt %), and PDMA-NMBA (30 wt %) were selected as preferred hydrogel systems going forward, with the aim to investigate a range of monomers and crosslinkers (Figures 4 and S10). While PDMAEMA was initially considered in this study to show the breadth of hydrogels that can be generated through the PET-RAFT approach, this polymer has previously been reported as cytotoxic and was, therefore, not considered for cell encapsulation.^65−67^ Cells were added to a mixture of monomer, crosslinker, CTA, and EY reconstituted in DMEM to reach a final cell density of 3 × 10^6^ cells mL^–1^ in a 500 μL total volume. From this suspension, 200 μL were collected and irradiated with blue light to obtain cell-laden hydrogels.

Cell viability within the cell-laden hydrogels was assessed with Live/Dead assay kit. For each system investigated, a viability of over 95% could be observed, demonstrating the high cytocompatibility of the PET-RAFT polymerization approach for the preparation of hydrogel scaffolds. Moreover, high cell viability (>90%) was maintained after incubating the hydrogel scaffolds for 7 days in cell culture media (Figure S11). The high cytocompatibility is likely a consequence of the high monomer conversion these systems can reach, which ensures no free monomer remains within the 3D network.

### Self-Healing

**Figure 5:**
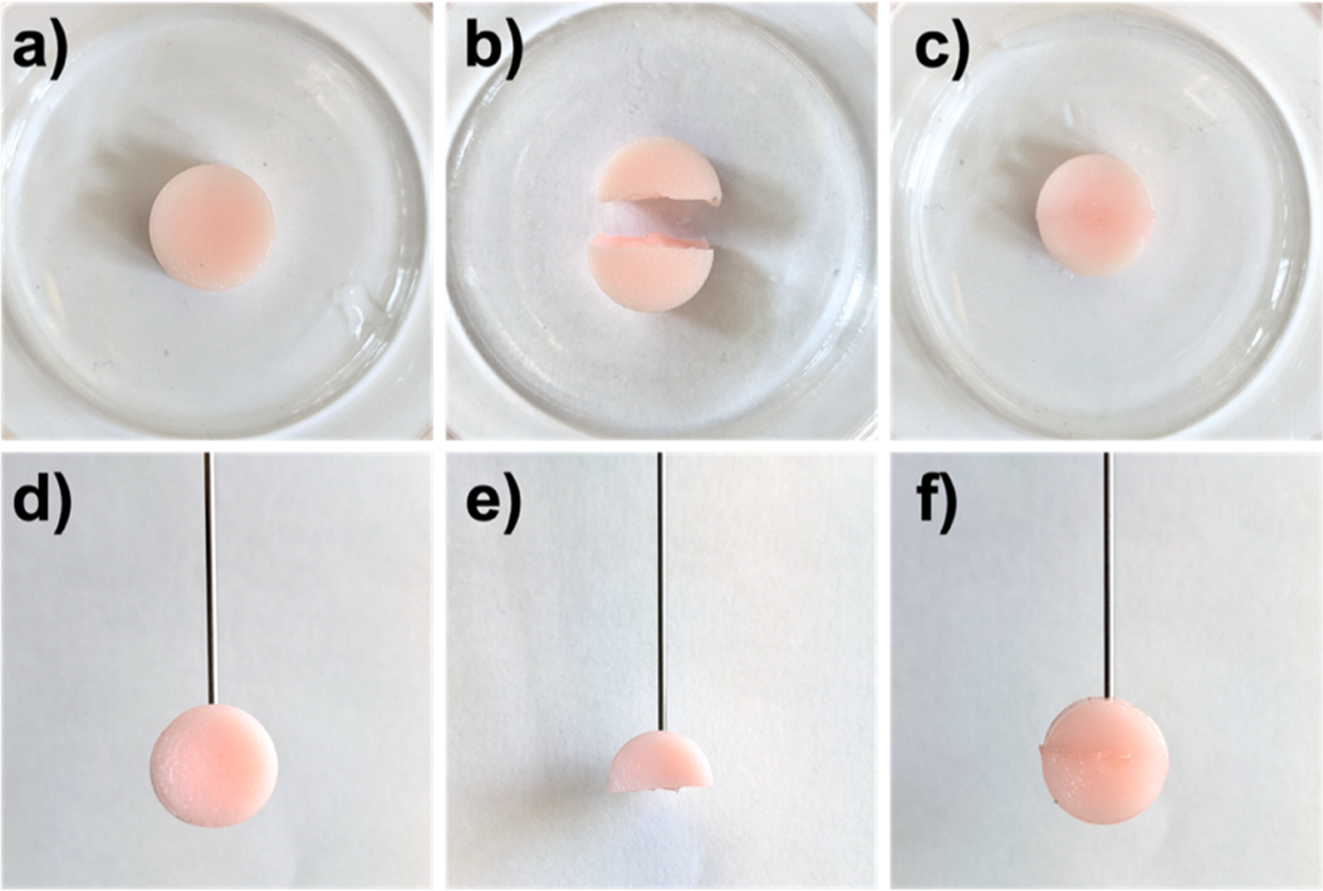
PET-RAFT polymerized hydrogels showing self-healing properties. PEGDA (DP 50) hydrogel as made (a) and cut in half (b). (c) Healed hydrogel after blue light irradiation for 60 min. PEGDA (DP 50) hydrogel as made suspended through a needle (d), cut in half (e), healed and capable of sustaining its own weight (f).

We hypothesized that the presence of the CTA at the end of each polymer chain and remaining EY within the hydrogel matrix could enable further chain propagation even after formation of the 3D network, affording hydrogels able to self-heal and repair after damage. To assess this, a PEGDA (DP 50) hydrogel was cut in half with a scalpel and both halves placed within proximity of each other, without full contact being made. PEGDA monomer (100 μL) was then added to fill the gap and the hydrogel irradiated for 60 min (Figure 5). The resultant healed hydrogel was able to support its own weight (Figure 5f) when suspended through a needle, showing no separation occurred between the two glued hydrogel blocks. When a control experiment was carried out in which no extra monomer was added to fill the gap between the hydrogel blocks, no healing was observed, and the individual pieces remained separate (Figure S12). The mechanical properties of the healed hydrogels were then assessed and compared to those of hydrogels prior to cutting (Figure S13 and Table S4). The PEGDA DP 50 hydrogel prepared with the higher EY concentration was used in this case, as a result of the better mechanical performance observed for this system compared to the PEGDA DP 100 hydrogel. As expected, the mechanical properties slightly decreased after self-healing (Table S4), with the Young’s modulus decreasing to 273.8 kPa (from 360.9 kPa) and the stress at break to 105.0 kPa (from 138.2 kPa). However, healed materials still showed good mechanical performance, demonstrating the ability of hydrogels prepared through a PET-RAFT approach to repair once damaged.

**Figure 6:**
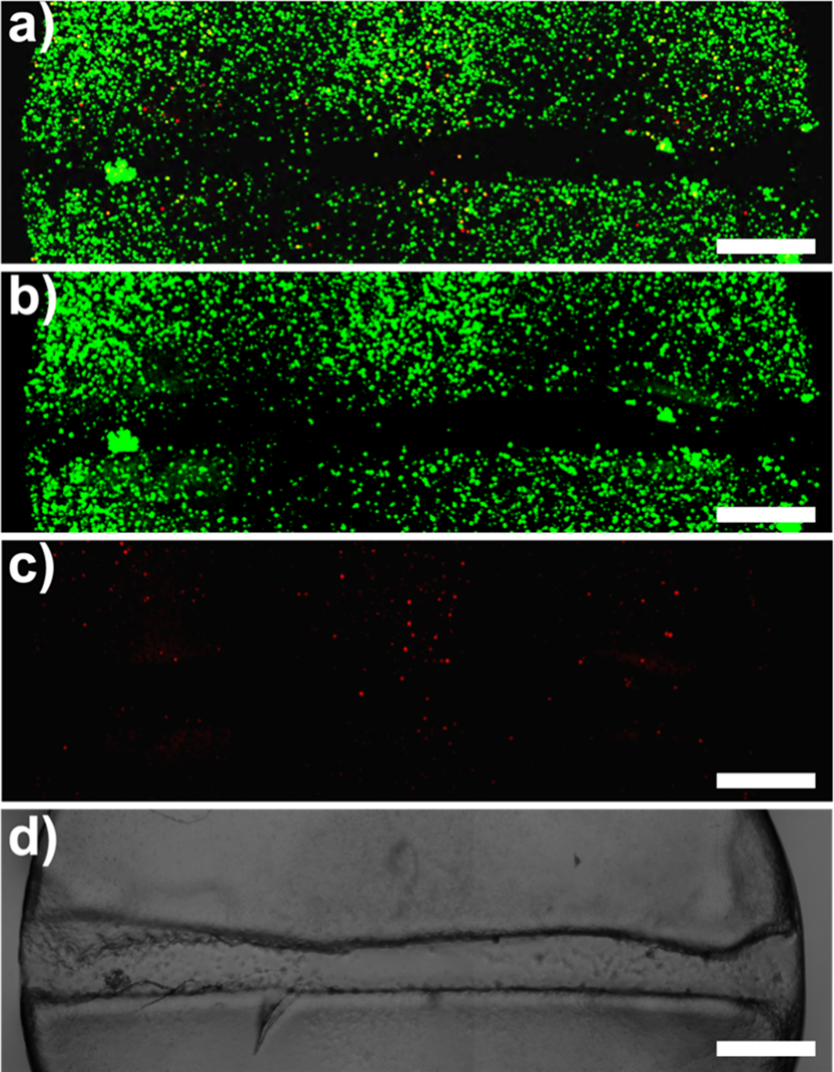
Overlay (a), live (b), dead (c), and brightfield (d) channels of healed PEGDA (DP 50) hydrogel encapsulating HPCs. Scale bar = 200 μm.

Following the successful self-healing in PBS, a similar experiment was designed for PEGDA (DP 50) hydrogels encapsulating living cells, adding a further monomer layer in the gap resulting from cutting the material in half. As expected, the hydrogel showed healing capability, with cells retaining high viability at the healed interface (Figure 6).

## Conclusions

In this work, we report a novel approach for the fabrication of hydrogels directly within cytocompatible media using PET-RAFT polymerization. Exploring a range of monomers and crosslinkers, hydrogels were obtained with high monomer conversions, good swelling properties, and mechanical performance. Our approach resulted in highly cytocompatible cell scaffolds, where cells could survive over an extended period (7 days) following encapsulation, owing to the high monomer conversions attained. Finally, we demonstrated the self-healing ability of our hydrogels, a process that can be exploited thanks to the activation/deactivation process typical of PET-RAFT polymerization. This work expands the scope of PET-RAFT polymerization into the fabrication of hydrogel materials, demonstrating the potential of this technique as a promising route for designing cytocompatible scaffolds for cell encapsulation. Moreover, this strategy opens the door to generate scaffolds for tissue engineering with varied chemical composition and mechanical properties by polymerizing and healing together hydrogels prepared from a range of different monomers, achieving a mechanical gradient typical of layered tissues.

## Supporting Information Available

Additional experimental details for hydrogel formulations, including exact molar ratios of each component and photographs of experimental setup; ^1^H NMR spectra showing conversion for all systems; additional swelling, rheological, and mechanical data for all systems, and additional cell viability images and mechanical analysis after self-healing (PDF)
