# Supplementary material for: Self-Healing Hydrogel Scaffolds through PET-RAFT Polymerization in Cellular Environment
Source: Biomacromolecules. 2023 Jun 29;24(7):3370–9. doi: 10.1021/acs.biomac.3c00431 (PMC10336845; doi:10.1021/acs.biomac.3c00431)
Supplement: Supplementary file 1 — bm3c00431_si_001.pdf [file bm3c00431_si_001.pdf]

Self-healing hydrogel scaffolds through PET-RAFT polymerization in cellular environment

Alasdair D. M. Rigby[†], *Amaziah R. Alipio*[†], *Viviane Chiaradia*[†], *Maria C. Arno*[*, †, §]

[†] *School of Chemistry, University of Birmingham, Edgbaston, Birmingham, B15 2TT, United Kingdom*

[§] *Institute of Cancer and Genomic Sciences, University of Birmingham, Edgbaston, Birmingham, B15 2TT, United Kingdom*

Table S1: Hydrogels prepared in this work, including targeted DP and ratios of monomer, crosslinker, EY, and CTA used. The percentage in brackets refers to the amount of crosslinker used in relation to monomer.

Hydrogel formulation	Targeted DP	Monomer (mmol)	Crosslinker (mmol)	CTA (mmol, 10^{-3})	EY (mmol, 10^{-5})
PEGDA	50	0.26	N/A	5.23	2.95
PEGDA	50	0.26	N/A	5.23	4.97
PEGDA	100	0.26	N/A	2.62	1.47
PEGMA-PEGDA (5 wt%)	100	0.42	1.3×10^{-2}	4.17	2.35
PEGMA-PEGDA (10 wt%)	100	0.42	2.6×10^{-2}	4.17	2.35
PDMAEMA-PEGDA (50 wt%)	100	0.95	13×10^{-2}	9.54	5.39
PDMA-NMBA (30 wt%)	100	1.01	19.5×10^{-2}	10.1	5.7
PDMA-NMBA (50 wt%)	100	1.01	32.4×10^{-2}	10.1	5.7

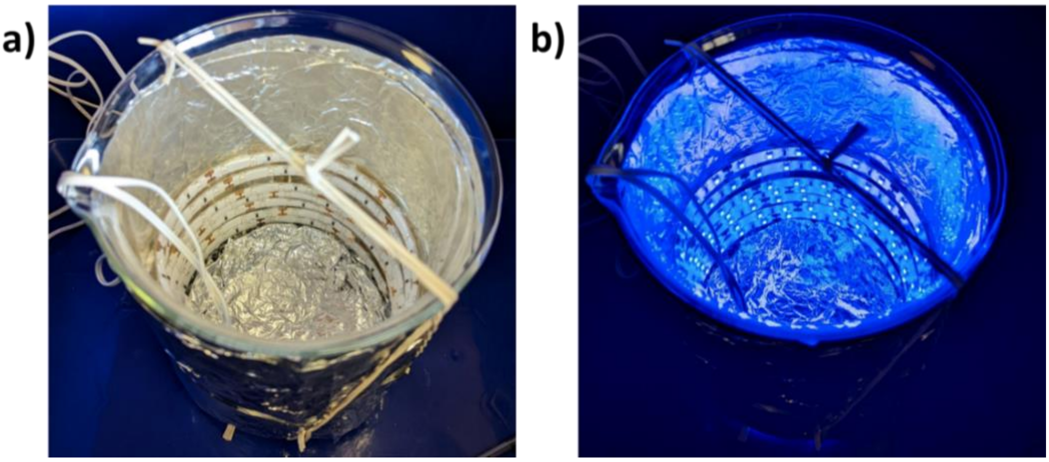

Figure S1: Photoreactor setup a) without irradiation and b) during irradiation, providing 11.5 W at 450 nm.

Table S2: Cell-laden hydrogels prepared in this work. The percentage in brackets refers to the amount of crosslinker used in relation to monomer.

Hydrogel	Targeted DP	Monomer (mmol)	Crosslinker (mmol)	CTA (mmol, 10^{-3})	EY (mmol, 10^{-5})	DMEM (µL)	HPC suspension in DMEM (µL)
PEGDA	50	0.26	N/A	5.23	2.95	337	163
PEGMA-PEGDA (5 wt%)	100	0.42	1.3×10^{-2}	4.17	2.35	335	165
PDMA-NMBA (30 wt%)	100	1.01	19.5×10^{-2}	10.1	5.7	345	155

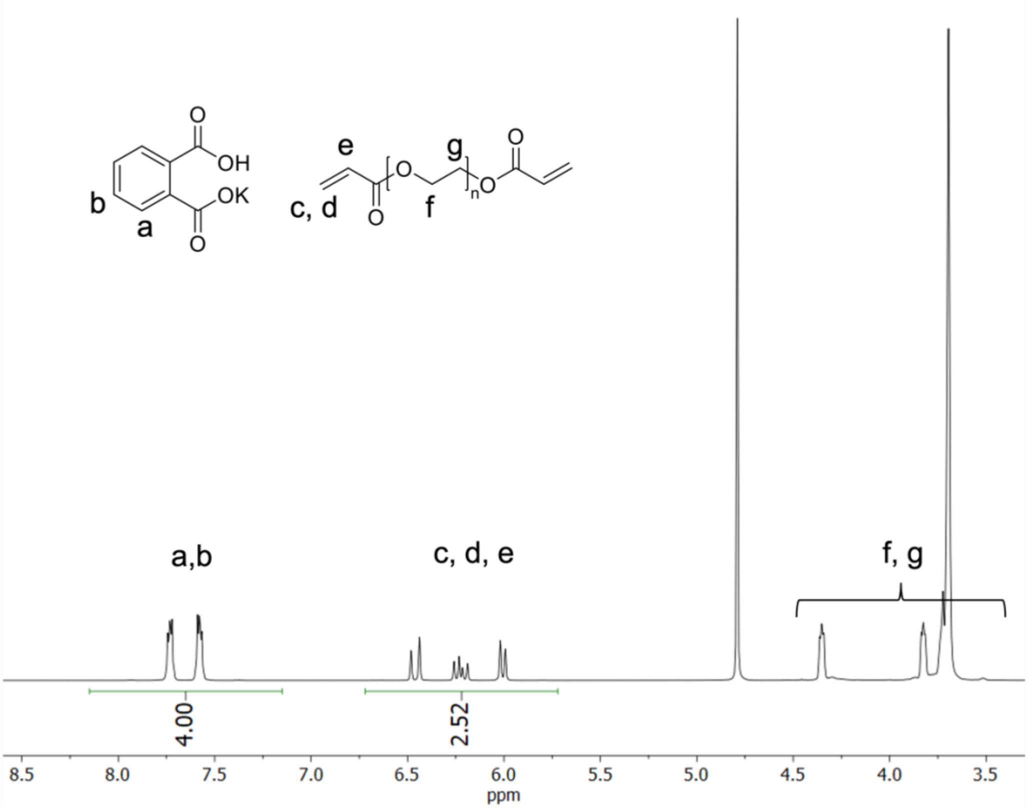

Figure S2: Representative ^{1}H NMR spectrum in D_2O (400 MHz, 298 K) used to calculate monomer conversion from a PEGDA (DP 50) hydrogel, showing the remaining monomer and internal calibrant peaks.

Table S3: Monomer conversion (C) and EWC of PEGDA (DP 50) hydrogels prepared using 9.51 mM (51 ppm) of EY. Values are reported as average ± standard deviation, where N = 3.

Hydrogel formulation	Targeted DP	C[a] (%)	EWC (%)
PEGDA	50	96 ± 1.1	80 ± 0.1

[a]Monomer conversions were calculated *via* qNMR spectroscopy against PHP as an internal standard.

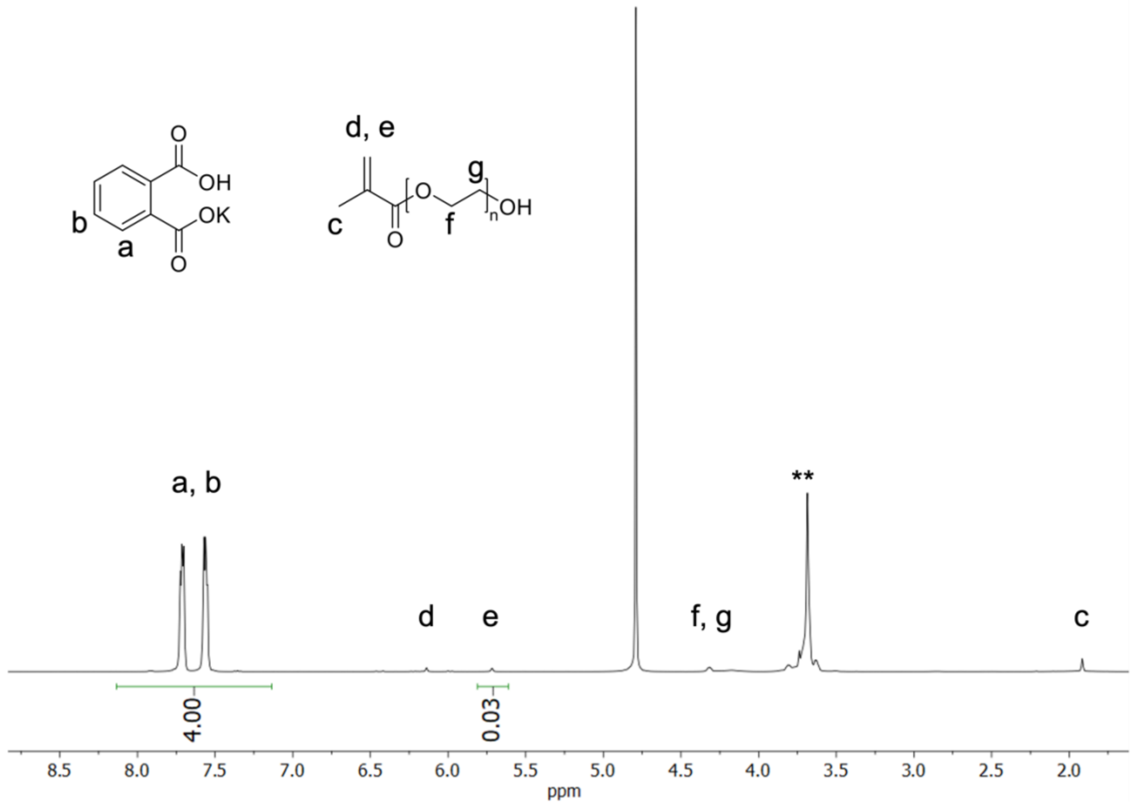

Figure S3: Representative ^{1}H NMR spectrum in D_2O (400 MHz, 298 K) used to calculate monomer conversion from a PEGMA-PEGDA (5 wt% of crosslinker) hydrogel, showing the remaining monomer and internal calibrant peaks. ** = PEGMA-PEGDA polymer in solution.

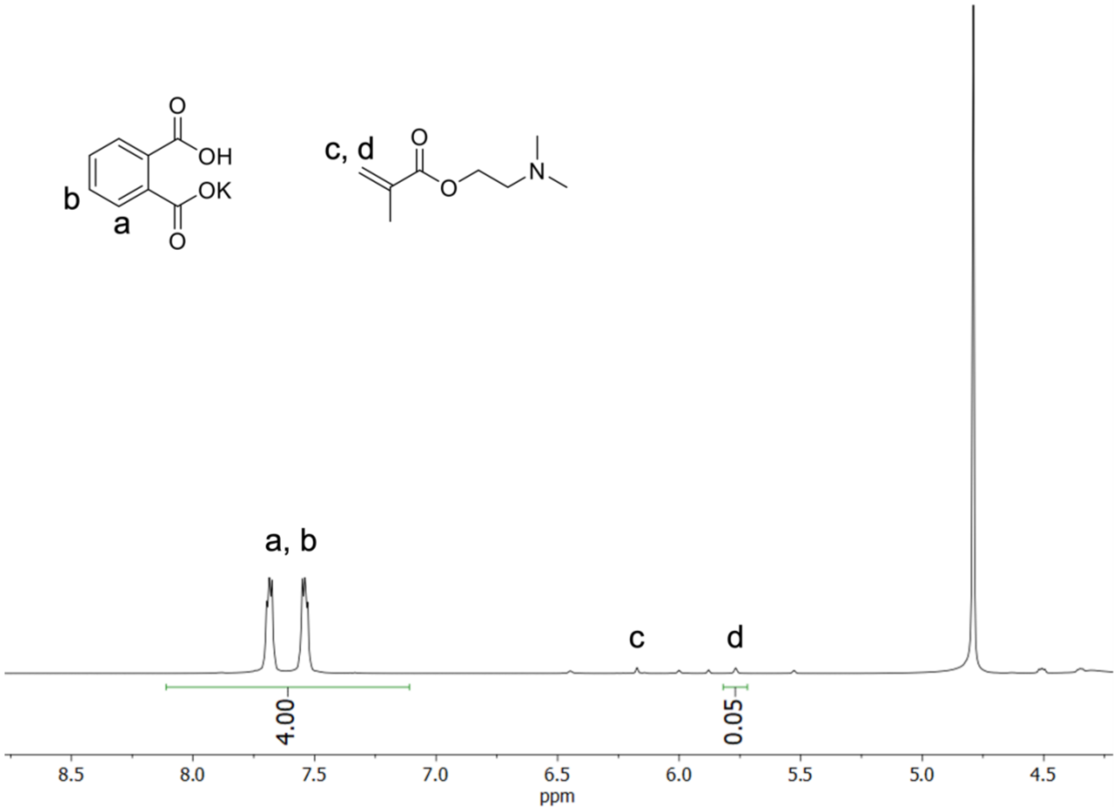

Figure S4: Representative ^{1}H NMR spectrum in D_2O (400 MHz, 298 K) used to calculate monomer conversion from a PDMAEMA-PEGDA (50 wt% of crosslinker) hydrogel, showing the remaining monomer and internal calibrant peaks.

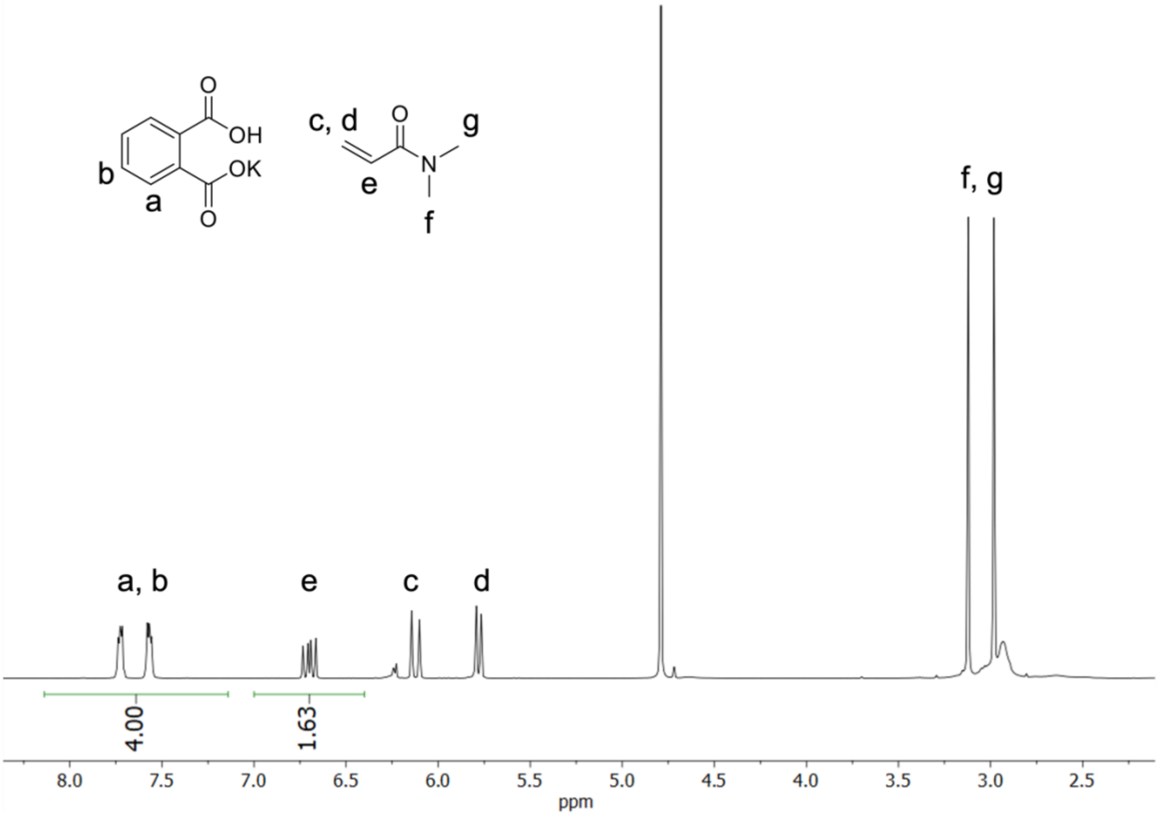

Figure S5: Representative ^{1}H NMR spectrum in D_2O (400 MHz, 298 K) used to calculate monomer conversion from a PDMA-NMBA (30 wt% of crosslinker) hydrogel, showing the remaining monomer and internal calibrant peaks.

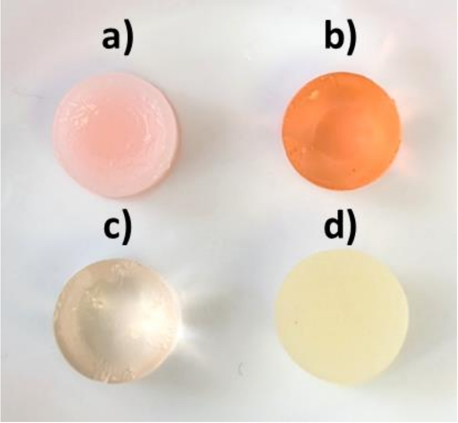

Figure S6: Hydrogels prepared in this work. a) PEGDA (DP 50); b) PEGMA-PEGDA (5 wt% of crosslinker); c) PDMA-NMBA (30 wt% of crosslinker); d) PDMAEMA-PEGDA (50 wt% of crosslinker).

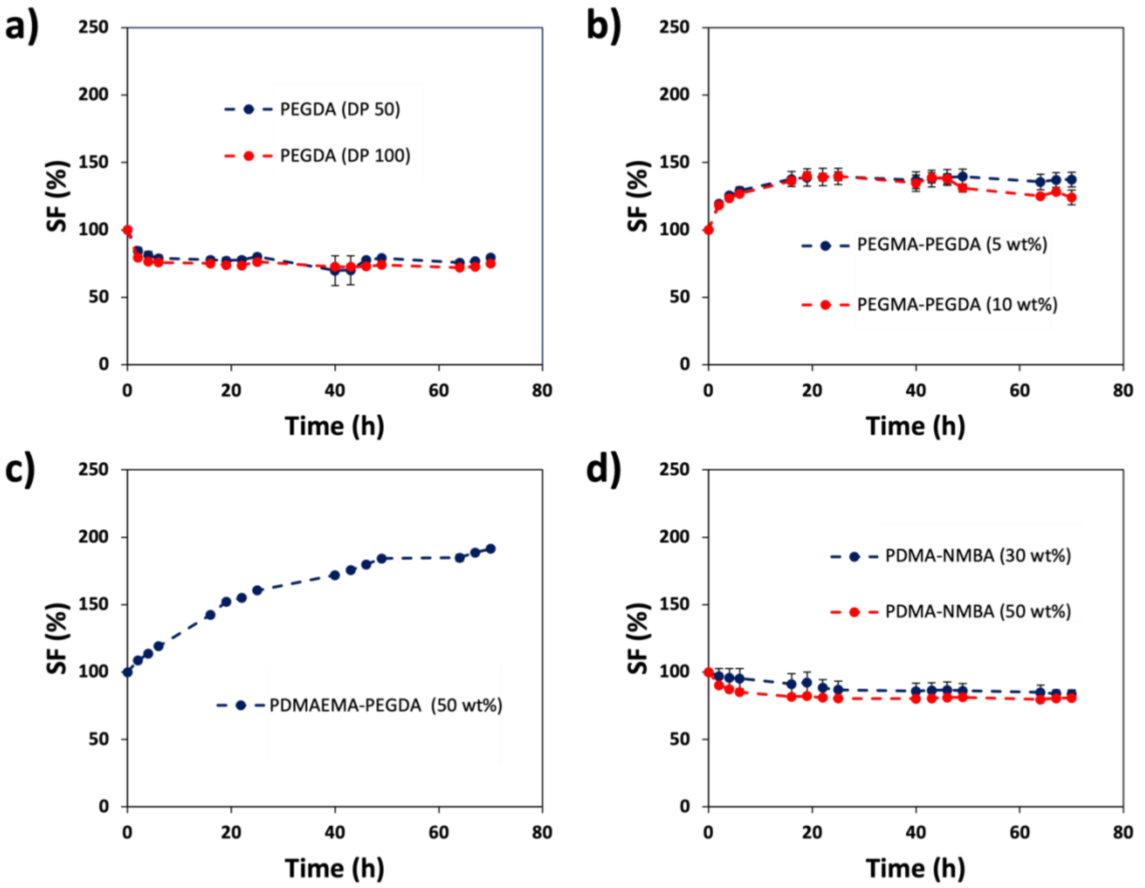

Figure S7: Characterization of hydrogel's swelling properties. Swelling factor (SF) (%) as a function of time in PBS solution at 37 °C.

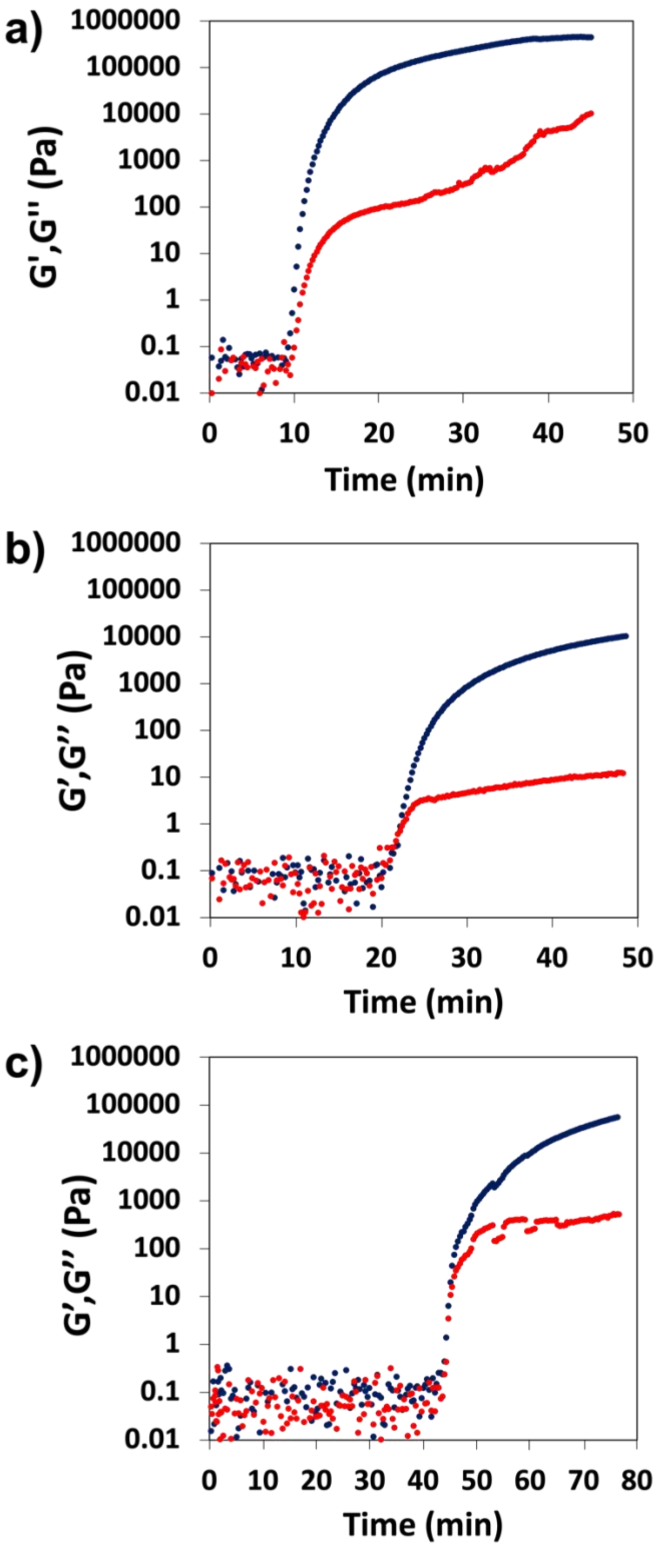

Figure S8: Photo-rheology of a) PEGDA (DP 100), b) PEGMA-PEGDA (10 wt%), and c) PDMA-NMBA (50 wt%) hydrogels showing the storage modulus (*G’*, red line) and loss modulus (*G’’*, blue line) *vs* irradiation time.

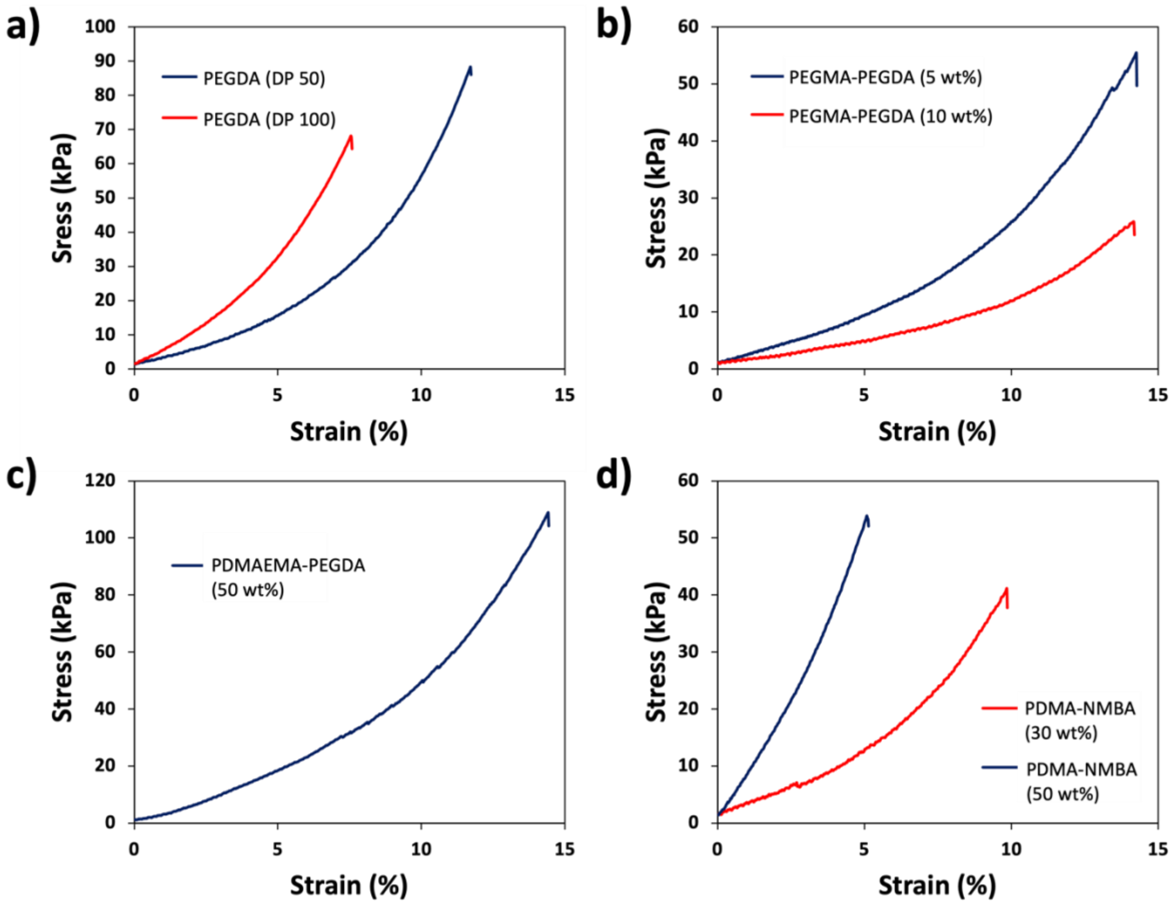

Figure S9: Mechanical testing analysis for hydrogels prepared in this work showing representitve stress *vs* strain curves for each system. a) PEGDA; b) PEGMA-PEGDA; c) PDMAEMA-PEGDA, d) PDMA-NMBA.

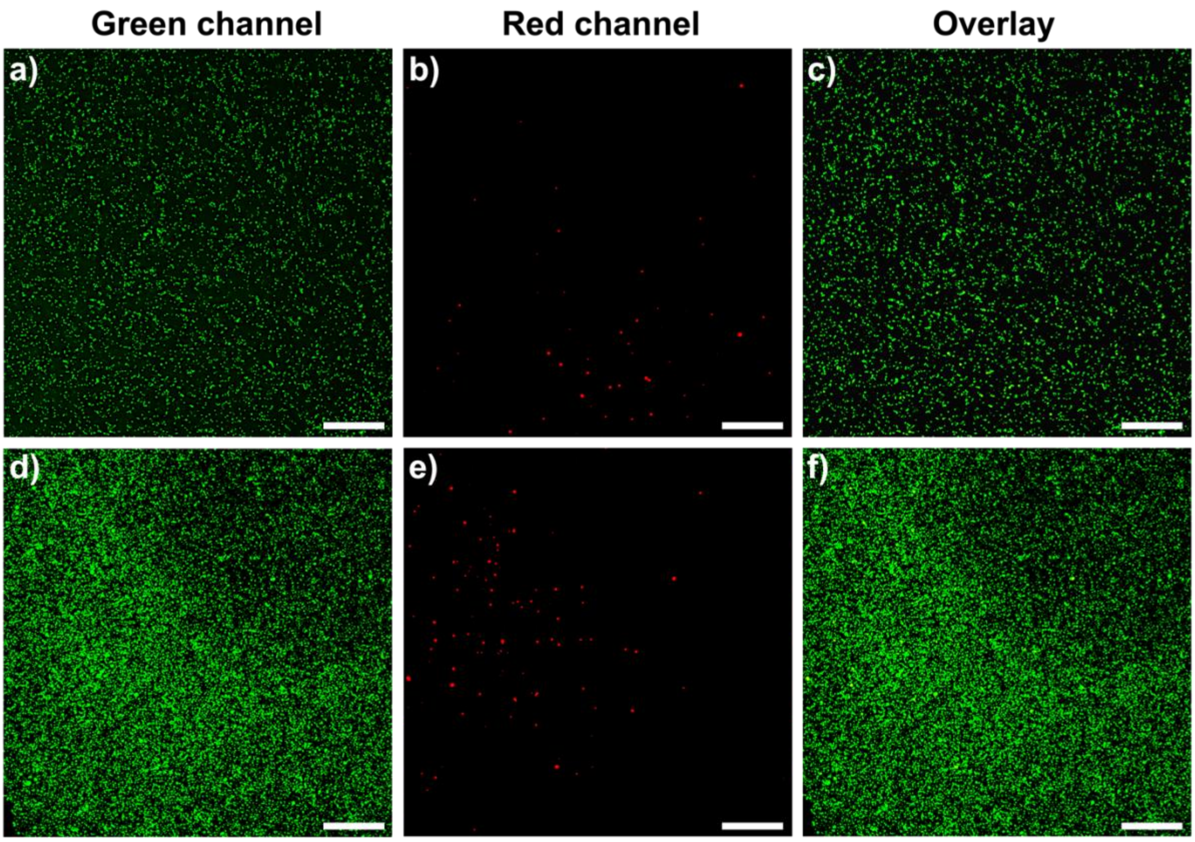

Figure S10: Cell viability of cell-laden hydrogels of PEGMA-PEGDA (5 wt%) (a, b, and c) and PDMA-NMBA (30 wt%) (d, e, and f). HPCs were stained with live/dead cell viability kit comprising of calcein-AM and ethidium-homodimer fluorescent dyes indicating live and dead cells indicated in green and red, respectively. Scale bar = 500 μm.

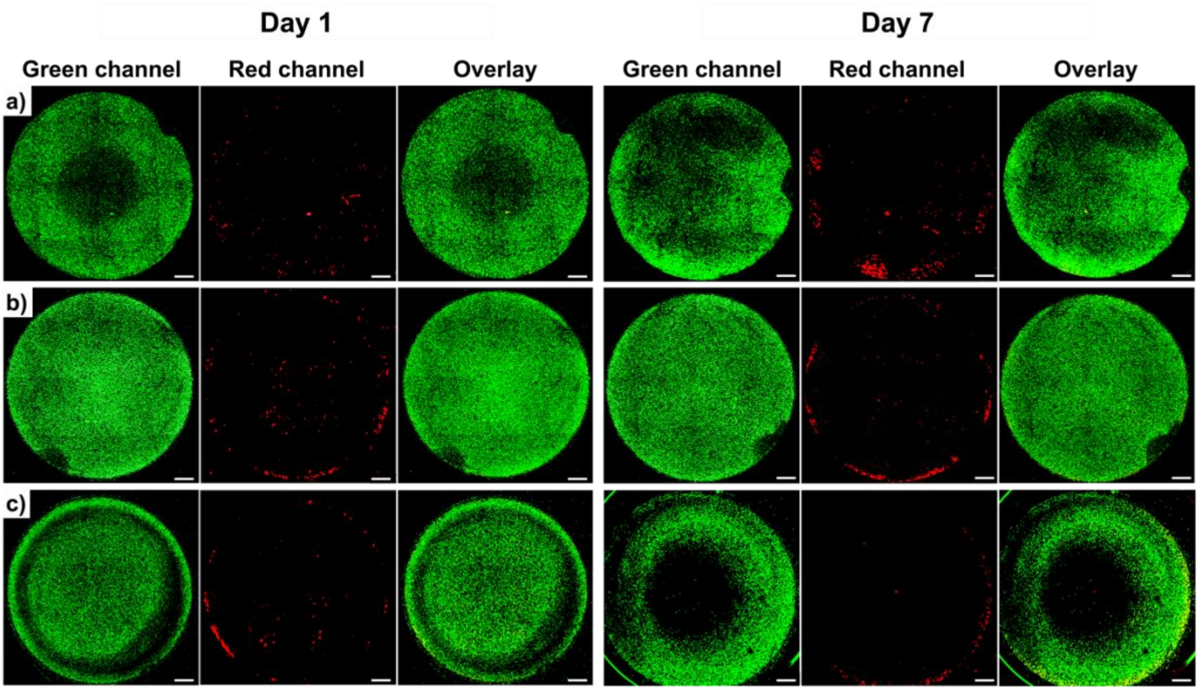

Figure S11: Cell viability of cell-laden hydrogels at day 1 and day 7. Confocal representative z-stack images of a) PDMA-NMBA (30 wt%), b) PEGDA (DP 50), and c) PEGMA-PEGDA (5 wt%) with each row representing cell-laden hydrogels captured at 1 day and 7 days post-encapsulation. HPCs were stained with live/dead cell viability kit comprising of calcein-AM and ethidium-homodimer fluorescent dyes indicating live and dead cells indicated in green and red, respectively. Z-stacks were captured at a depth of > 500 µm at 10x magnification and stitched using Olympus CellSens software. Scale bar = 1 mm.

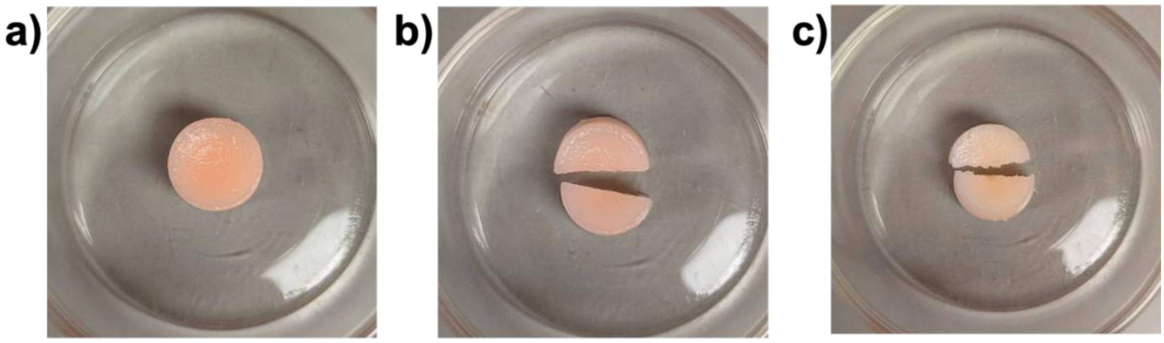

Figure S12: Control self-healing experiment. PEGDA (DP50) hydrogel as made (a) and cut in half (b). c) Cut hydrogel after piecing both halves back together and irradiating with blue light for 1 h without addition of extra monomer, showing lack of healing.

Table S4: Mechanical testing analysis for PEGDA (DP50) hydrogels prepared with higher concentration of EY (9.51 mM) before and after healing, showing average compressive Young's modulus, strain at break, and stress at break.

Hydrogel formulation	Young's modulus, E (kPa)	Strain at break (%)	Stress at break (kPa)
PEGDA (DP 50) as prepared	360.9 ± 109.0	10.5 ± 2.6	138.2 ± 75.5
PEGDA (DP 50) after healing	273.8. ± 37.4	10.0 ± 1.6	105.0 ± 37.4

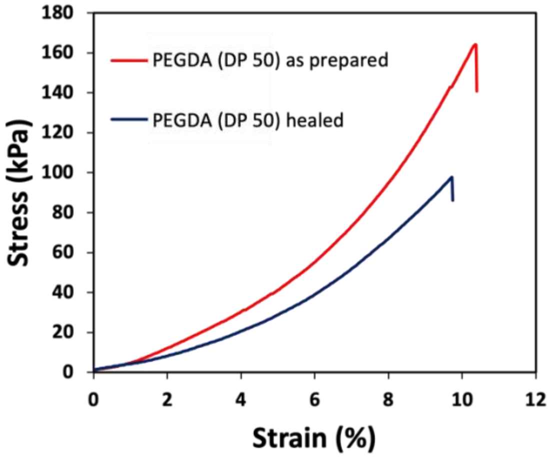

Figure S13: Mechanical testing analysis showing representitve stress *vs* strain graphs for PEGDA (DP 50) hydrogels prepared with higher concentration of EY (9.51 mM) before and after healing.
